# Association of Maternal and Paternal Astigmatism With Child Astigmatism in the Hong Kong Children Eye Study

**DOI:** 10.1001/jamanetworkopen.2022.47795

**Published:** 2022-12-21

**Authors:** Ka Wai Kam, Arnold S. H. Chee, Yuzhou Zhang, Rex C. Y. Tang, Jason T. C. Chan, Xiu Juan Zhang, Yu Meng Wang, Ryan C. F. Chan, Wai Kit Chu, Patrick Ip, Alvin L. Young, Clement C. Tham, Chi Pui Pang, Li Jia Chen, Jason C. Yam

**Affiliations:** 1 Department of Ophthalmology and Visual Sciences, The Chinese University of Hong Kong, Hong Kong Eye Hospital, Hong Kong SAR, China; 2 Department of Ophthalmology and Visual Sciences, Prince of Wales Hospital, Hong Kong SAR, China; 3 Hong Kong Hub of Paediatric Excellence, The Chinese University of Hong Kong, Hong Kong SAR, China; 4 Department of Paediatrics and Adolescent Medicine, LKS Faculty of Medicine, The University of Hong Kong, Hong Kong SAR, China; 5 Hong Kong Eye Hospital, Kowloon, Hong Kong SAR, China; 6 Department of Ophthalmology, Hong Kong Children’s Hospital, Hong Kong SAR, China

## Abstract

**Importance:**

Parental astigmatism is a factor associated with risk for development of child astigmatism; however, the magnitude of the association has not been determined.

**Objective:**

To determine the association between parental and child astigmatism.

**Design, Setting, and Participants:**

This population-based, cross-sectional study included participants from familial trios, each comprising a child aged 6 to 8 years and both parents, recruited from the Hong Kong Children Eye Study. No restriction criteria were set on the children in terms of refractive status. Data were analyzed from February to June 2022.

**Exposures:**

Cycloplegic autorefraction and autokeratometry were conducted on the children, whereas noncycloplegic autorefraction and autokeratometry were conducted on their parents. The children were categorized into 6 groups on the basis of the severity of astigmatism of both parents. Information on parental education, family income, and children’s outdoor and near work time were obtained by questionnaires.

**Main Outcomes and Measures:**

The primary outcome was the odds of child astigmatism among the 6 categories of children. Associations of factors with child astigmatism were evaluated by logistic regression analyses.

**Results:**

A total of 17 124 participants from 5708 trios (2964 boys and 2754 girls) at a mean (SD) age of 7.32 (0.87) years, and 11 416 parents were examined. Astigmatism of 1.0 D or greater in both parents was associated with greater odds of refractive astigmatism (RA) (odds ratio [OR], 1.62; 95% CI, 1.15-2.26) and corneal astigmatism (CA) (OR, 1.94; 95% CI, 1.50-2.50) in the child. The respective ORs increased to 3.10 (95% CI, 1.34-7.21) and 4.31 (95% CI, 1.76-10.55) when both parents had astigmatism 2.0 D or greater. Higher parental astigmatism conferred higher risks for both RA and CA in children (*P* for trend <.001). Parental astigmatism was significantly associated with greater odds of corresponding child astigmatism (maternal RA: OR, 0.76; 95% CI, 0.68-0.84; paternal RA: OR, 0.82; 95% CI, 0.74-0.91; maternal CA: OR, 1.70; 95% CI, 1.51-1.93; paternal CA: OR, 1.33; 95% CI, 1.19-1.49).

**Conclusions and Relevance:**

The findings of this cross-sectional study suggest that parental astigmatism may confer an independent and dose-dependent association with child astigmatism. Children with parents with astigmatism should have early eye examinations for timely detection of astigmatism to facilitate age-appropriate vision correction and visual development.

## Key Points

**Question:**

What is the association between parental astigmatism and child astigmatism?

**Findings:**

In this population-based cross-sectional study that included 5708 children and 11 416 parents, among other reported biological, environmental, and sociodemographic factors, parental astigmatism was shown to be the most important factor associated with risk of child astigmatism. Both parents were associated wth the inheritance of astigmatism.

**Meaning:**

These findings suggest that early screening for astigmatism should be targeted at children of parents with high levels of astigmatism to provide timely detection and intervention to prevent amblyopia.

## Teaser

This cross-sectional study evaluates the association of maternal and paternal astigmatism with child astigmatism in the Hong Kong Children Eye Study.

## Introduction

Astigmatism is a common refractive error worldwide, with a global prevalence of 14.9% in children and 40.4% in adults.^1^ Uncorrected astigmatism can lead to amblyopia. The Vision in Preschoolers study^2^ on children aged from 3 to 5 years in the US reported that 13.2% of eyes with untreated astigmatism of 1.0 D or greater and less than 2.0 D were amblyopic, and so were 19.6% of eyes with untreated astigmatism of 2.0 D or greater. Furthermore, astigmatism is associated with myopia development.^3,4,5^ Higher degrees of myopia in eyes with greater than 1.0 D astigmatism were observed in a cohort of 298 children aged 10 years or younger.^3^ Moreover, in 108 Hong Kong Chinese preschoolers aged 3 to 6 years, higher degrees of astigmatism exhibited a greater myopic shift and increased axial length after 5 years.^5^ Unlike myopia or hyperopia, for which visual focus can be attained respectively at near distance or with accommodative effort, astigmatism prevents clear vision at any distance, disrupting the contrast sensitivity and spatial processing of stimuli.^6^ These visual deficits can lead to disruptive effects in cognitive functions, language ability, and fine motor tasks. Children aged 1 to 3 years with 2.0 D or greater uncorrected astigmatism did worse in cognitive and language scales according to the Bayley Scales of Infant and Toddler Development (3rd edition, BSITD-III).^7^

The cause of astigmatism is complex and not yet fully understood. Major risk factors include genetics, myopic refraction, and Asian ethnicity.^8,9^ In particular, parental association with astigmatism is inconsistent in different studies. The Beaver Dam Study found significant parent-child associations for myopia and hyperopia among 225 parent-children pairs, but not for astigmatism.^10^ Teikari et al^11^ studied 20 twin pairs recruited from the Finnish Twin Cohort Study and suggested that genetic factors had no association with on inducing astigmatism after adjusting for environmental factors. Conversely, upon evaluating 506 female British twin pairs, using data from both eyes in a multivariate model, Hammond et al^12^ identified a dominant genetic association in astigmatism and estimated that heritability for astigmatism ranged from 50% to 65%, lower than the 86% quoted for myopia or hyperopia. The 2002 Tehran Eye Study^13,14^ constructed conditional models involving 3806 participants to estimate the odds ratios (ORs) for astigmatism among parent-child pairs, and found ORs of 1.35 for total astigmatism, 1.53 for with-the-rule astigmatism (ie, the cornea is steeper vertically), and 2.13, for against-the-rule astigmatism (ie, the cornea is steeper horizontally), all lower than the ORs of 1.82 to 3.81 for myopia in the same study. Notably, although genome-wide association studies (GWAS)^15,16,17^ have identified several loci associated with astigmatism, data from population-based cross-sectional studies gave differing evidence. For instance, the Sydney Myopia Study^18^ on 468 children and their parents who were using spectacle prescriptions found no significant difference in mean cylinder values between children with parents with astigmatism and those with parents with astigmatism.

Astigmatism has been observed to be more prevalent among Chinese children when compared with other populations.^19,20^ On the basis of refractive errors and high prevalence of refractive astigmatism (RA) and corneal astigmatism (CA) observed in our cohort of 9281 Hong Kong Chinese school children and adults,^21^ we speculate a positive parental association with astigmatism in children. To determine the quantitative associations between parental and child astigmatism, we recruited trios, each comprising a child and both parents. We investigated the prevalence of astigmatism in children with parents who were nonastigmatic and astigmatic, and evaluated the associations between parental and child astigmatisms, taking into account the child’s age, sex, ocular parameters, environmental factors, and parental education and income levels.

## Methods

### Study Participants

Participants for this cross-sectional study were recruited from the Hong Kong Children Eye Study, an ongoing population-based cohort study on eye conditions in children aged 6 to 8 years recruited from primary schools in Hong Kong.^22,23,24,25,26^ All parents provided written informed consent before participating, and the study conformed to the tenets of the Declaration of Helsinki.^27^ Ethics approval was obtained from the institutional review board of the Chinese University of Hong Kong. This report followed the Strengthening the Reporting of Observational Studies in Epidemiology (STROBE) reporting guideline. The details of our methods, including the inclusion and exclusion criteria, have been published previously.^24,26^ In brief, school children aged 6 to 8 years were eligible and randomly sampled from 7 cluster regions determined by the Hong Kong Government according to the population distribution. No restriction criteria were set regarding the refractive status or visual acuity of the child. Parents who had keratorefractive or intraocular surgery, or corneal pathology were identified from a questionnaire and excluded. All children and their parents underwent complete ophthalmoscopic examinations and provided information about their socioeconomic status, demographic and health-related data, and environmental factors associated with risk for astigmatism through standardized questionnaires. We established a trio study by nonselectively recruiting families with both parents attending eye examination. Families with only 1 parent completing assessment tasks, families with twins or multiple children, and families with parents who had prior keratorefractive surgery for any refractive errors were excluded from the trio analyses.

### Ocular Examinations

All children were measured for RA using an autorefractor unit (Nidek ARK-510A) following 2 cycles of 1% cyclopentolate (Cyclogyl, Alcon-Convreur) and 1% tropicamide eye drops (Santen) administered 10 minutes apart. If a pupillary light reflex remained, or the pupil size remained less than 6 mm, a third cycle of eye drops would be administered. At least 3 readings of spherocylindrical autorefraction were obtained 30 minutes after the last dose of cycloplegic agents and then averaged. All parents were measured for noncycloplegic refraction. Lastly, children and parents were measured for CA using the autokeratometer (Nidek ARK-510A). The magnitude of CA was calculated as the difference between the steepest and flattest meridians, while the axis of CA was defined as the meridian of the flattest curvature.

### Definitions of Astigmatism

Astigmatism was defined using absolute values with a threshold of 1.0 D. RA was expressed using negative notation and defined as astigmatism of –1.0 D or less. Mild RA was defined as RA between –1.0 and –2.0 D, and high RA as –2.0 D or greater. CA was expressed using positive notation and defined as astigmatism of 1.0 D or greater. Mild CA was defined as CA greater than or equal to 1.0 D to less than 2.0 D, and high CA was defined as as 2.0 D or greater.^28^ To affirm high correlation between the right and left eyes, we compared the astigmatism between the 2 eyes (eTable 1 and eTable 2 in Supplement 1), and a paired Wilcoxon signed-rank test demonstrated no significant interocular difference. Consequently, our analyses used only measurements from the participants’ right eyes. Sensitivity analyses were performed using the left eyes, mean of both eyes, and worse eyes (eTable 4 in Supplement 1).

### Questionnaires on Parental Education Level, Children’s Outdoor Time, and Near Work

The validated questionnaires used in our study were derived from the Chinese version of the Sydney Myopia Study (eAppendix in Supplement 1).^18,28^ Parents completed the questionnaires with assistance from our trained staff, either in person or via telephone. If data were missing from questionnaire responses, our staff contacted parents by telephone for follow-up.

### Statistical Analysis

Demographic information and clinical characteristics were summarized using descriptive statistics. Continuous variables were reported in means and SDs, while categorical variables were reported in frequencies and percentages. Prevalence values and 95% CIs were calculated for both RA and CA. Univariate logistic regression models were used to estimate the associations of parental astigmatism and other factors associated with risk of child astigmatism. Parental astigmatism was first modeled separately as maternal and paternal astigmatisms for any independent association with child astigmatism. The β coefficients for maternal and paternal astigmatism were compared for a difference in the association. Then, parental astigmatism was evaluated as a categorical variable, where children were classified into 6 parental groups according to the astigmatism severity among their parents. The second model aimed to assess any dose-dependent association between the number and severity of parents with astigmatism and the risk of child astigmatism. Multiple logistic regression models were used to estimate the risk of child astigmatism in each subgroup, adjusted for child’s age, sex, spherical equivalent/axial length (AL), outdoor time, near work time, family income, and parent’s education level. *P* values for trends of parental association were further estimated by univariate analysis. *P* < .05 was considered significant. All analyses were performed using SPSS version 24.0 (IBM). Data were analyzed February to June 2022.

## Results

### Prevalence of Astigmatism Among Children and Parents

The mean (SD) age for the 5708 children (2954 boys and 2754 girls) in this trio study was 7.32 (0.87) years (range, 6-8 years) (Table 1), while for the parents, it was 41.54 (5.75) years. The mean (SD) spherical equivalent was 0.23 (1.54) D and the mean (SD) axial length (AL) was 23.03 (0.93) mm in children. The RA prevalence was 24.2% (95% CI, 23.1%-25.3%) and CA prevalence was 60.5% (95% CI, 59.2%-61.8%) among the children, while among parents the respective prevalences were 33.1% (95% CI, 32.3%-34.0%) and 43.4% (95% CI, 42.4%-44.3%). The prevalences of child RA and CA increased when both parents were astigmatic and with a greater severity of parental RA or CA.

**Table 1.** Demographic Characteristics of the 5708 Trios Included in the Study

Characteristic	Mean (SD)
Child	
Age, y	7.32 (0.87)
Sex, No. (%)	
Male sex	2954 (51.8)
Female	2754 (48.2)
Ocular examination values	
Refractive astigmatism, D	−0.73 (0.66)
Corneal astigmatism, D	1.25 (0.67)
Spherical equivalent, D	0.23 (1.54)
Axial length, mm	23.03 (0.93)
Outdoor time, h/d	1.40 (0.53)
Near work, D h/d	9.22 (2.68)
Maternal	
Age, y	39.77 (4.60)
Postsecondary education, No. (%)	2494 (43.7)
Ocular examination values	
Refractive astigmatism, D	−0.79 (0.69)
Corneal astigmatism, D	1.07 (0.69)
Paternal	
Age, y	43.29 (6.22)
Postsecondary education, No. (%)	2803 (49.1)
Ocular examination values	
Refractive astigmatism, D	−0.90 (0.78)
Corneal astigmatism, D	1.03 (0.73)
Family income per month,<HK $20 000, No. (%)^a^	1261 (22.1)

Abbreviation: h/d, hours per day.

^a^ HK $20 000 is equivalent to US $2555.61 as of November 18, 2022.

### Associations Between Parental Astigmatism Among Other Factors and Child Astigmatism

Univariate analyses found that both maternal and paternal astigmatism, sex, and child spherical equivalent values were associated with both child RA and CA (Table 2). In particular, female sex was associated with a lesser likelihood of RA (odds ratio [OR], 0.87; 95% CI, 0.77-0.99; *P* = .03) and a greater likelihood of CA (OR, 1.19; 95% CI, 1.07-1.32; *P* = .002). A longer AL was associated with a lower odds of child CA in the univariate analysis (OR, 0.76; 95% CI, 0.71-0.80; *P* < .001). However, there were no associations of sex with child CA and RA after adjusting for parental astigmatism (eTable 3 in Supplement 1 and Table 3).

**Table 2.** The Association of Parental Astigmatism and the Other Factors Associated with Risk for Child Astigmatism by Univariate Regression Models in 5708 Trios

Factor	OR (95% CI)^a^	*P* value
Child refractive astigmatism		
Refractive astigmatism, D		
Maternal	0.74 (0.69-0.81)	<.001
Paternal	0.82 (0.76-0.88)	<.001
Child’s age, y	0.97 (0.90-1.04)	.37
Female sex	0.87 (0.77-0.99)	.03
Child’s spherical equivalent, D	0.87 (0.84-0.91)	<.001
Outdoor time, h/d	0.93 (0.80-1.07)	.30
Near work , D h/d	1.00 (0.98-1.03)	.79
Family income per month,<HK $20 000 as reference	0.96 (0.81-1.14)	.67
Postsecondary education level, without postsecondary as reference		
Mother	0.98 (0.85-1.13)	.75
Father	0.90 (0.78-1.04)	.14
Child corneal astigmatism		
Corneal astigmatism, D		
Maternal	1.75 (1.60-1.91)	<.001
Paternal	1.29 (1.20-1.40)	<.001
Child’s age, y	0.94 (0.89-1.00)	.06
Female sex	1.19 (1.07-1.32)	.002
Child’s axial length, mm	0.76 (0.71-0.80)	<.001
Outdoor time, h/d	0.88 (0.78-1.00)	.05
Near work, D h/d	1.01 (0.99-1.04)	.37
Family income per month,<HK $20 000 as reference	1.11 (0.96-1.29)	.16
Postsecondary education level, without postsecondary as reference		
Mother	0.90 (0.80-1.02)	.11
Father	0.87 (0.77-0.98)	.03

Abbreviations: h/d, hours per day; OR, odds ratio.

^a^ OR was estimated by univariate logistic regression model.

**Table 3.** The Association of Parental Astigmatism and Other Factors Associated With Risk on Child Astigmatism by Multiple Regression Models in 5708 Trios

Factor	OR (95% CI)^a^	*P* value
Child refractive astigmatism		
Refractive astigmatism, D		
Maternal	0.76 (0.68-0.84)	<.001
Paternal	0.82 (0.74-0.91)	<.001
Child’s age, years	0.90 (0.81-1.00)	.04
Sex, male as reference	0.88 (0.75-1.04)	.13
Child’s spherical equivalent, D	0.86 (0.82-0.91)	<.001
Outdoor time, h/d	0.92 (0.78-1.08)	.31
Near work, D h/d	1.00 (0.96-1.03)	.75
Family income per month,<HK $20 000 as reference	0.94 (0.76-1.17)	.57
Postsecondary education level, without postsecondary as reference		
Mother	1.03 (0.84-1.27)	.77
Father	0.85 (0.69-1.05)	.12
Child corneal astigmatism		
Corneal astigmatism, D		
Maternal	1.66 (1.47-1.88)	<.001
Paternal	1.38 (1.24-1.54)	<.001
Child’s age, years	1.06 (0.96-1.16)	.26
Sex, male as reference	0.93 (0.79-1.09)	.37
Child’s axial length, mm	0.76 (0.69-0.83)	<.001
Outdoor time, h/d	0.89 (0.77-1.02)	.10
Near work, D h/d	1.01 (0.98-1.04)	.45
Family income per month,<HK $20 000 as reference	1.15 (0.94-1.40)	.18
Postsecondary education level, without postsecondary as reference		
Mother	0.99 (0.82-1.19)	.92
Father	0.83 (0.69-1.00)	.05

Abbreviations: h/d, hours per day; OR, odds ratio.

^a^ OR was estimated by multiple logistic regression model.

### Parental Astigmatism as an Independent Factor Associated With Risk for Child Astigmatism

In the multiple regression models, the associations between parental and child astigmatism remained significant after adjustments for the child’s sex, spherical equivalent, outdoor time, near work time, family income, and parent’s education levels (Table 3). Myopic spherical equivalent was associated with RA (OR, 0.86; 95% CI, 0.82-0.91; *P* < .001) and the child’s AL was associated with CA (OR, 0.76; 95% CI, 0.69-0.83; *P* < .001) after adjusting for parental astigmatism, environmental and demographic factors. Older age of children was also consistently associated with less RA (OR, 0.90; 95% CI, 0.81-1.00; *P* = .04) but not with CA (OR, 1.06; 95% CI, 0.96-1.16; *P* = .26). Finally, the child’s outdoor time, near work time, family income, and parental education levels were not associated with either RA or CA (Table 3).

A dose-dependent association exists for both types of astigmatism as the prevalence and odds of child astigmatism increased with the number of parents with astigmatism (Table 4 and Table 5). Only 1 parent having mild RA conferred no significant association on child RA. The risk became significant when both parents had mild RA (OR, 1.62; 95% CI, 1.15-2.26; *P* = .01) and increased when both parents had high RA (OR, 3.10; 95% CI, 1.34-7.21; *P* < .001). On the other hand, only 1 parent having mild CA was already associated with higher odds of CA in the child (OR, 1.47; 95% CI, 1.23-1.75; *P* < .001). The risk of child CA increased by nearly 2-fold if both parents had mild CA (OR, 1.94; 95% CI, 1.50-2.50; *P* < .001) and by more than 4-fold if both parents had high CA (OR, 4.31; 95% CI, 1.76-10.55; *P* = .001) regardless of the age, sex, axial length, outdoor time and near work time of the child, parental education levels, and family income. Significant trends were also observed between parental astigmatisms and the respective risks for child RA and child CA (*P* for trend <.001) (Table 5 and eTable 5 and eFigure in Supplement 1).

**Table 4.** Prevalence of Child Astigmatism in Different Combinations of Parental Astigmatism Severities in 5708 Trios

Parental astigmatism status	Children, No.	Age of children, mean (SD), y	Prevalence of child astigmatism, % (95% CI)
Parental refractive astigmatism status			
No astigmatism plus no astigmatism	2530	7.35 (0.87)	21.6 (20.0-23.2)^a^
No astigmatism plus mild astigmatism^b^	1904	7.32 (0.87)	22.5 (20.6-24.4)^a^
Mild astigmatism plus mild astigmatism	343	7.31 (0.86)	27.6 (23.0-32.7)^a^
High astigmatism plus no astigmatism	654	7.22 (0.87)	31.5 (28.0-35.3)^a^
High astigmatism plus mild astigmatism	221	7.31 (0.88)	37.3 (30.9-44.0)^a^
High astigmatism plus high astigmatism	41	7.27 (0.79)	43.9 (28.5-60.3)^a^
Overall	5693	7.32 (0.87)	24.2 (23.1-25.3)^a^
Parental corneal astigmatism status			
No astigmatism plus no astigmatism	1785	7.39 (0.87)	51.0 (48.7-53.4)^c^
No astigmatism plus mild astigmatism	2101	7.32 (0.87)	61.1 (59.0-63.3)^c^
Mild astigmatism plus mild astigmatism	623	7.33 (0.86)	67.0 (63.2-70.8)^c^
High astigmatism plus no astigmatism	551	7.26 (0.86)	67.6 (63.5-71.5)^c^
High astigmatism plus mild astigmatism	382	7.17 (0.86)	77.1 (72.5-81.2)^c^
High astigmatism plus high astigmatism	51	7.22 (0.90)	84.0 (70.9-92.8)^c^
Overall	5493	7.33 (0.87)	60.5 (59.2-61.8)^c^

^a^ Prevalence of child refractive astigmatism.

^b^ This group contained trios of mothers without astigmatism plus fathers with mild astigmatism, as well as trios of fathers without astigmatism plus mothers with mild astigmatism. The same is applied to all 6 groups.

^c^ Prevalence of child corneal astigmatism.

**Table 5.** The Association of Parental Astigmatism on Child Astigmatism in 5708 Trios

Parental refractive astigmatism status	Result (95% CI)^a^	*P* value
Child refractive astigmatism, OR^b^		
No astigmatism plus no astigmatism	1 [Reference]	NA
No astigmatism plus mild astigmatism^c^	1.03 (0.85 to 1.25)	.78
Mild astigmatism plus mild astigmatism	1.62 (1.15 to 2.26)	.01
High astigmatism plus no astigmatism	1.55 (1.19 to 2.01)	.001
High astigmatism plus mild astigmatism	2.22 (1.52 to 3.24)	<.001
High astigmatism plus high astigmatism	3.10 (1.34 to 7.21)	.01
Child refractive astigmatism, estimated mean D^b^		
No astigmatism plus no astigmatism	−0.67 (−0.71 to −0.63)	NA
No astigmatism plus mild astigmatism	−0.72 (−0.76 to −0.67)	NA
Mild astigmatism plus mild astigmatism	−0.80 (−0.90 to −0.71)	NA
High astigmatism plus no astigmatism	−0.82 (−0.89 to −0.75)	NA
High astigmatism plus mild astigmatism	−0.95 (−1.06 to −0.83)	NA
High astigmatism plus high astigmatism	−1.08 (−1.34 to −0.81)	NA
*P* value for trend^d^	NA	<.001
Child corneal astigmatism, OR^a^^,^^e^		
No astigmatism plus no astigmatism	1 [Reference]	NA
No astigmatism plus mild astigmatism	1.47 (1.23 to 1.75)	<.001
Mild astigmatism plus mild astigmatism	1.94 (1.50 to 2.50)	<.001
High astigmatism plus no astigmatism	1.87 (1.43 to 2.44)	<.001
High astigmatism plus mild astigmatism	4.20 (2.90 to 6.08)	<.001
High astigmatism plus high astigmatism	4.31 (1.76 to 10.55)	.001
Child corneal astigmatism, estimated mean D^e^		
No astigmatism plus no astigmatism	1.11 (1.06 to 1.15)	NA
No astigmatism plus mild astigmatism	1.21 (1.16 to 1.25)	NA
Mild astigmatism plus mild astigmatism	1.35 (1.28 to 1.42)	NA
High astigmatism plus no astigmatism	1.41 (1.34 to 1.49)	NA
High astigmatism plus mild astigmatism	1.62 (1.53 to 1.71)	NA
High astigmatism plus high astigmatism	1.68 (1.46 to 1.90)	NA
*P* value for trend^d^	NA	<.001

Abbreviations: NA, not applicable; OR, odds ratio.

^a^ OR was estimated by multiple logistic regression model.

^b^ Age, sex, child’s spherical equivalent, outdoor time, near work, family income, and parental education level were adjusted in refractive astigmatism model.

^c^ This group contained trios of mothers without astigmatism plus fathers with mild astigmatism, as well as trios of fathers without astigmatism plus mothers with mild astigmatism. The same is applied to all 6 groups.

^d^ *P* value for trend of parental association was estimated by univariate analysis.

^e^ Age, sex, child’s axial length, outdoor time, near work, family income, and parental education level were adjusted in corneal astigmatism model.

### Associations With Parental, Maternal, and Paternal Astigmatism

Parental astigmatism demonstrated the greatest association with child astigmatism among all factors included in the multiple logistic regression (Table 3). Parental astigmatism was significantly associated with greater odds of corresponding child astigmatism (maternal RA OR, 0.76; 95% CI, 0.68-0.84; paternal RA OR, 0.82; 95% CI, 0.74-0.91; maternal CA OR, 1.70; 95% CI, 1.51-1.93; paternal CA OR, 1.33; 95% CI, 1.19-1.49). The magnitudes of associations were greater in maternal and paternal CA (adjusted OR [aOR], 1.66; 95% CI, 1.47-1.88; *P* < .001 for maternal CA, and aOR, 1.38; 95% CI, 1.24-1.54; *P* < .001 for paternal CA) than those observed in RA. However no significant difference was detected in terms of the associations between maternal and paternal CA or RA with child CA or RA (β = 0.51 vs β = 0.32 and β = −0.28 vs β = −0.20).

## Discussion

To our knowledge, this is the first population-based, cross-sectional trio study to establish parental astigmatism as an independent factor associated with risk for both refractive and corneal astigmatism among children. In 5708 trios involving 17 124 study participants, the risk follows a dose-dependent association after adjustments for the child’s age, sex, spherical equivalent, near work time, outdoor time, family income, and parental education levels. Children with parents with astigmatism should therefore be provided early screening to detect astigmatism and to prescribe necessary spectacle correction for prevention of amblyopia development.

In this study, we have affirmed the association of parental astigmatism with child astigmatism in Chinese parent-child trios living in the highly urbanized city of Hong Kong. Although twin-based studies and GWASs have identified genetic associations for CA and RA, evidence of a hereditary effect has been limited and inconclusive. For instance, in the Sydney Myopia Study,^26^ although there was a higher prevalence of astigmatism among 183 children aged 6 and 12 years with parents with astigmatism compared with 285 children with parents without astigmatism, such differences did not reach significance. The majority of children were White, and the overall prevalence of astigmatism (6.4%) was low when compared with Chinese children, having only been detected in 30 of 468 children.^26^ Conversely, there is a higher prevalence of astigmatism for both Chinese children and adults. The RA (≤–1.0 D) prevalence was 21.9% and CA (≥1.0 D) prevalence 63.9% among Chinese children, and the respective prevalence among Chinse adults was 30.9% and 39.5%.^29^ High prevalence may be due to genetic effects. However, the genetic basis of the mechanism and ethnic differences of astigmatism have not been established. In this study, the use of actual measurement data eliminated potential bias or misclassification arising from spectacle prescription, while the sample size of over 5700 trios is sufficiently large to allow examination of only the right eyes without needing statistical adjustments to increase power. To the best of our knowledge, this is the first study to quantify the associations of parental astigmatism with child CA and RA.

We have determined a dose-dependent association between parental and child astigmatisms. The astigmatic status of both parents contributes to astigmatism development in their child. In this study, the prevalence of both RA and CA in children increased with both the number of parents with astigmatism and the severity of parental astigmatism. In particular, the greater association observed for CA further supports inheritability for CA, which was determined solely by the steep and flat corneal curvatures in this study. This reflects findings from GWASs, which have identified specific candidate genes for CA, but not for RA.^15,16,30^ Meta-analyses from the CREAM Consortium^15,16^ identified only 1 genome-wide significant locus near the platelet-derived growth factor receptor α gene with a pooled OR of 1.12 (95% CI, 1.08-1.16) for CA, and none for RA. Similarly, the magnitudes of associations were greater in parental CA than those of parental RA. We speculate that CA is more physiological in its cause, but RA can be caused by other physiological and multiple environmental, or external factors. Nevertheless, the parental association remained significant, likely because the cornea contributes primarily to ocular astigmatism.

Results of our study also quantified the magnitude of association between parental and child astigmatism (Table 4), which can serve as a clinical guide for counseling parents with astigmatism, in addition to allowing health care workers to better triage resources for screening preschool children of parents with astigmatism for uncorrected astigmatism. This is impactful to the public health of populations with a high prevalence, such as Chinese people. Previous population-based studies^8,31,32^ have identified child spherical equivalent as a major risk factor for child astigmatism. Although our data support this association with child RA, we also showed greater associations between parental astigmatism and both child RA and CA. Regarding other factors associated with risk, although parental education levels were previously thought to contribute to a myogenic environment for children,^33^ our data concur with a population-based survey in Korea,^34^ which showed that it did not pose additional risks for child astigmatism. On the other hand, given that outdoor time reduces the risk of myopia, and that myopic children are more likely to have astigmatism, the Nanjing Eye Study^35^ inferred that more outdoor activity time acted as a protective mechanism to increase the amount of oblique internal astigmatism compensation, thus lowering the risk of astigmatism. In our study, also in Chinese children, greater outdoor time was marginally associated with lower CA, yet the association did not remain after adjusting for parental astigmatism.

### Limitations

Findings of our study need to be interpreted with the following limitations. First, the questionnaires surveying the outdoor and near work time of the child were prone to recall bias. Using wearable sensors to measure outdoor and near work time in future studies may help to provide more objective data and reduce misclassification. Second, the current phase of the Hong Kong Children Eye Study is a cross-sectional study, which can only determine associations but not causation. We are currently collecting longitudinal follow-up data for further analysis and future report. Third, although we conducted strict cycloplegic protocols on the children to ensure accurate refraction, we only managed to use noncycloplegic autorefraction and autokeratometry on their parents. This may constitute a potential source of bias to the study, as autorefraction measurements may differ after cycloplegia. The use of noncycloplegic refraction in adults may overestimate myopic parameters such as spherical equivalent and spherical values. However, none of these parameters were included in this study as a major variable. A difference in the vertical component of astigmatism had been shown following cycloplegia; however, the actual magnitude of difference is trivial by a mean of 0.01 D in J_0_ and none in the oblique component (J_45_).^36^ As our study only evaluated the magnitude of overall astigmatism instead of individual components, the effect of noncycloplegic measurements on overall astigmatism magnitude should be minimal. Additionally, our study examined Chinese children within a defined age range and parents from Hong Kong; thus, the findings may not be generalized to a broader population.

## Conclusions

In conclusion, this cross-sectional study found that both parental RA and CA had independent, dose-dependent associations with the occurrence of astigmatism in children. Parents with astigmatism should receive counseling on increased risks of astigmatism in their children, and early screening should be offered to high-risk children for timely detection and interventions.

## Notes

Supplement 1. **eTable 1.** Comparison of Astigmatism in Right Eye and Left Eye **eTable 2.** Table of Contingencies for Left and Right Eyes **eTable 3.** The Association of Parental Astigmatism on Child Astigmatism in 5708 Trios Using Different Cutoff Points for Astigmatism **eTable 4.** The Association of Parental Astigmatism on Child Astigmatism in 5708 Trios Using Data from Different Eyes **eTable 5.** Age and Sex Association Analysis on Child Astigmatism in 5708 Trios **eFigure.** Different Combinations of Parental Astigmatism Severities on Child Astigmatism **eAppendix.** Hong Kong Children Eye Study Questionnaire

Supplement 2.
Data Sharing Statement
