# Supplementary material for: Association of Maternal and Paternal Astigmatism With Child Astigmatism in the Hong Kong Children Eye Study
Source: JAMA Netw Open. 2022 Dec 21;5(12):e2247795. doi: 10.1001/jamanetworkopen.2022.47795 (PMC9856878; doi:10.1001/jamanetworkopen.2022.47795)
Supplement: Supplement 1. — eTable 1. Comparison of Astigmatism in Right Eye and Left Eye eTable 2. Table of Contingencies for Left and Right Eyes eTable 3. The Association of Parental Astigmatism on Child Astigmatism in 5708 Trios Using Different Cutoff Points for Astigmatism eTable 4. The Association of Parental Astigmatism on Child Astigmatism in 5708 Trios Using Data from Different Eyes eTable 5. Age and Sex Association Analysis on Child Astigmatism in 5708 Trios eFigure. Different Combinations of Parental Astigmatism Severities on Child Astigmatism eAppendix. Hong Kong Children Eye Study Questionnaire [file jamanetwopen-e2247795-s001.pdf]

Supplemental Online Content

Kam KW, Chee ASH, Zhang Y, et al. Association of maternal and paternal astigmatism with child astigmatism in the Hong Kong Children Eye Study. *JAMA Netw Open*. 2022;5(12):e2247795.
doi:10.1001/jamanetworkopen.2022.47795

eTable 1. Comparison of Astigmatism in Right Eye and Left Eye
eTable 2. Table of Contingencies for Left and Right Eyes
eTable 3. The Association of Parental Astigmatism on Child Astigmatism in 5708 Trios Using Different Cutoff Points for Astigmatism
eTable 4. The Association of Parental Astigmatism on Child Astigmatism in 5708 Trios Using Data from Different Eyes
eTable 5. Age and Sex Association Analysis on Child Astigmatism in 5708 Trios
eFigure. Different Combinations of Parental Astigmatism Severities on Child Astigmatism
eAppendix. Hong Kong Children Eye Study Questionnaire

This supplemental material has been provided by the authors to give readers additional information about their work.

© 2022 Kam KW et al. *JAMA Network Open.*

eTable 1. Comparison of Astigmatism in Right Eye and Left Eye

	Right eye	Left eye
Child's refractive astigmatism, D	-0.72 (0.68)	-0.77 (0.70)
Child's corneal astigmatism, D	1.25 (0.66)	1.29 (0.69)
Child's refractive astigmatism		
No	4319 (75.8%)	4102 (72.6%)
Yes	1379 (24.2%)	1550 (27.4%)
Child's corneal astigmatism		
No	2179 (39.4%)	2045 (37.0%)
Yes	3345 (60.6%)	3484 (63.0%)
Maternal refractive astigmatism, D	-0.79 (0.69)	-0.82 (0.75)
Maternal corneal astigmatism, D	1.07 (0.69)	1.12 (0.72)
Maternal refractive astigmatism		
No	4031 (70.8%)	3907 (68.8%)
Mild	1259 (22.1%)	1273 (22.4%)
High	407 (7.1%)	501 (8.8%)
Maternal corneal astigmatism		
No	3050 (54.5%)	2879 (51.7%)
Mild	2019 (36.1%)	2059 (37.0%)
High	525 (9.4%)	632 (11.3%)
Paternal refractive astigmatism, D	-0.91 (0.78)	-0.91 (0.80)
Paternal corneal astigmatism, D	1.03 (0.73)	1.05 (0.74)
Paternal refractive astigmatism		
No	3594 (63.0%)	3558 (62.7%)
Mild	1558 (27.3%)	1501 (26.5%)
High	662 (9.7%)	613 (10.8%)
Paternal corneal astigmatism		
No	3283 (58.8%)	3170 (57.0%)
Mild	1771 (31.7%)	1842 (33.1%)
High	532 (9.5%)	552 (9.9%)

© 2022 Kam KW et al. *JAMA Network Open.*

eTable 2. Table of Contingencies for Left and Right Eyes

Child's Refractive Astigmatism		
Right eye \ Left eye	No	Yes
No	3782	473
Yes	292	1076

Child's Corneal Astigmatism		
Right eye \ Left eye	No	Yes
No	1534	627
Yes	476	2828

Maternal Refractive Astigmatism			
Right eye \ Left eye	No	Mild	High
No	3448	509	60
Mild	438	658	162
High	21	106	279

Maternal Corneal Astigmatism			
Right eye \ Left eye	No	Mild	High
No	2322	647	42
Mild	511	1254	242
High	21	154	343

Paternal Refractive Astigmatism			
Right eye \ Left eye	No	Mild	High
No	2903	592	73
Mild	606	747	201
High	49	162	339

Paternal Corneal Astigmatism			
Right eye \ Left eye	No	Mild	High
No	2562	626	43
Mild	537	1046	168
High	30	159	338

© 2022 Kam KW et al. *JAMA Network Open.*

eTable 3. The Association of Parental Astigmatism on Child Astigmatism in 5708 Trios Using Different Cutoff Points for Astigmatism

Child refractive astigmatism was defined as astigmatism of ≤–*0.75D*			Child corneal astigmatism was defined as astigmatism of ≥*0.75D*		
	Child Refractive Astigmatism			Child Corneal Astigmatism	
	OR[a] (95% CI)	*P* Value		OR[a] (95% CI)	*P* Value
Maternal refractive astigmatism, D	0.85 (0.76-0.93)	0.001	Maternal corneal astigmatism, D	1.61 (1.38-1.87)	<0.001
Paternal refractive astigmatism, D	0.84 (0.77-0.92)	<0.001	Paternal corneal astigmatism, D	1.39 (1.21-1.60)	<0.001
Child's age, years	0.94 (0.86-1.03)	0.16	Child's age, years	0.94 (0.84-1.05)	0.27
Sex, male as reference	0.87 (0.75-1.00)	0.05	Sex, male as reference	1.03 (0.86-1.25)	0.75
Child's spherical equivalent, D	0.92 (0.88-0.97)	0.001	Child's axial length, mm	0.81 (0.72-0.89)	<0.001
Outdoor time, hours per day	0.98 (0.85-1.13)	0.78	Outdoor time, hours per day	0.88 (0.75-1.05)	0.16
Near work, diopter hours per day	1.00 (0.97-1.03)	0.92	Near work, diopter hours per day	1.03 (0.99-1.06)	0.18
Family income per month, <HK $20000 as reference	1.04 (0.86-1.25)	0.71	Family income per month, <HK $20000 as reference	1.03 (0.81-1.30)	0.83
Mother education level, without post-secondary as reference	1.02 (0.85-1.21)	0.85	Mother education level, without post-secondary as reference	1.09 (0.87-1.35)	0.46
Father education level, without post-secondary as reference	0.86 (0.72-1.03)	0.10	Father education level, without post-secondary as reference	0.91 (0.73-1.13)	0.40
**Child refractive astigmatism was defined as astigmatism of ≤–*1.25D***			**Child corneal astigmatism was defined as astigmatism of ≥*1.25D***		
	**Child Refractive Astigmatism**			**Child Corneal Astigmatism**	
	OR[a] (95% CI)	*P* Value		OR[a] (95% CI)	*P* Value
Maternal refractive astigmatism, D	0.72 (0.64-0.80)	<0.001	Maternal corneal astigmatism, D	1.71 (1.53-1.92)	<0.001

© 2022 Kam KW et al. *JAMA Network Open.*

Paternal refractive astigmatism, D	0.83 (0.74-0.93)	0.001	Paternal corneal astigmatism, D	1.42 (1.28-1.58)	<0.001
Child's age, years	0.87 (0.77-0.97)	0.02	Child's age, years	1.03 (0.94-1.14)	0.48
Sex, male as reference	0.94 (0.77-1.14)	0.51	Sex, male as reference	0.91 (0.78-1.07)	0.26
Child's spherical equivalent, D	0.81 (0.77-0.87)	<0.001	Child's axial length, mm	0.75 (0.69-0.83)	<0.001
Outdoor time, hours per day	0.96 (0.80-1.16)	0.69	Outdoor time, hours per day	0.96 (0.83-1.11)	0.58
Near work, diopter hours per day	1.00 (0.96-1.04)	0.98	Near work, diopter hours per day	1.01 (0.98-1.04)	0.38
Family income per month, <HK $20000 as reference	0.99 (0.77-1.27)	0.92	Family income per month, <HK $20000 as reference	1.01 (0.83-1.23)	0.93
Mother education level, without post-secondary as reference	0.99 (0.78-1.26)	0.94	Mother education level, without post-secondary as reference	0.96 (0.80-1.16)	0.67
Father education level, without post-secondary as reference	0.85 (0.67-1.08)	0.19	Father education level, without post-secondary as reference	0.89 (0.74-1.07)	0.20

Abbreviation: D, diopter; OR, odds ratio; CI, confidence interval.

[a] OR was estimated by multiple logistic regression model.

© 2022 Kam KW et al. *JAMA Network Open.*

eTable 4. The Association of Parental Astigmatism on Child Astigmatism in 5708 Trios Using Data from Different Eyes

**Using Data from *Left Eye***					
**Child Refractive Astigmatism[c]**			**Child Corneal Astigmatism[d]**		
	OR[a] (95% CI)	*P* Value		OR[a] (95% CI)	*P* Value
Maternal refractive astigmatism, D	0.76 (0.69-0.85)	<0.001	Maternal corneal astigmatism, D	1.41 (1.26-1.57)	<0.001
Paternal refractive astigmatism, D	0.82 (0.74-0.90)	<0.001	Paternal corneal astigmatism, D	1.32 (1.18-1.48)	<0.001
Child's age, years	0.96 (0.87-1.05)	0.36	Child's age, years	1.09 (0.99-1.20)	0.08
Sex, male as reference	0.96 (0.82-1.13)	0.62	Sex, male as reference	0.89 (0.76-1.04)	0.15
Child's spherical equivalent, D	0.87 (0.83-0.92)	<0.001	Child's axial length, mm	0.77 (0.70-0.84)	<0.001
Outdoor time, hours per day	0.92 (0.79-1.08)	0.30	Outdoor time, hours per day	0.89 (0.77-1.03)	0.11
Near work, diopter hours per day	1.00 (0.97-1.03)	0.95	Near work, diopter hours per day	1.02 (0.99-1.05)	0.33
Family income per month, <HK $20000 as reference	1.13 (0.92-1.40)	0.25	Family income per month, <HK $20000 as reference	1.01 (0.83-1.24)	0.91
Mother education level, without post-secondary as reference	1.11 (0.91-1.35)	0.31	Mother education level, without post-secondary as reference	1.00 (0.83-1.20)	0.95
Father education level, without post-secondary as reference	0.71 (0.58-0.87)	0.001	Father education level, without post-secondary as reference	0.94 (0.78-1.13)	0.52
**Using Data from *Average of Both Eyes***					
**Child Refractive Astigmatism, D[c]**			**Child Corneal Astigmatism, D[d]**		
	OR[a] (95% CI)	*P* Value		OR[a] (95% CI)	*P* Value
Maternal refractive astigmatism, D	0.73 (0.65-0.82)	<0.001	Maternal corneal astigmatism, D	1.72 (1.51-1.94)	<0.001

© 2022 Kam KW et al. *JAMA Network Open.*

Paternal refractive astigmatism, D	0.78 (0.70-0.87)	<0.001	Paternal corneal astigmatism, D	1.39 (1.23-1.56)	<0.001
Child's age, years	0.92 (0.83-1.02)	0.10	Child's age, years	1.04 (0.95-1.14)	0.40
Sex, male as reference	0.93 (0.78-1.10)	0.40	Sex, male as reference	0.86 (0.74-1.01)	0.07
Child's spherical equivalent, D	0.84 (0.79-0.89)	<0.001	Child's axial length, mm	0.75 (0.69-0.83)	<0.001
Outdoor time, hours per day	0.90 (0.76-1.07)	0.24	Outdoor time, hours per day	0.84 (0.73-0.97)	0.02
Near work, diopter hours per day	1.00 (0.97-1.04)	0.93	Near work, diopter hours per day	1.02 (0.99-1.05)	0.30
Family income per month, <HK $20000 as reference	1.02 (0.81-1.27)	0.87	Family income per month, <HK $20000 as reference	1.02 (0.84-1.25)	0.81
Mother education level, without post-secondary as reference	0.96 (0.78-1.19)	0.73	Mother education level, without post-secondary as reference	0.98 (0.82-1.18)	0.83
Father education level, without post-secondary as reference	0.79 (0.64-0.98)	0.03	Father education level, without post-secondary as reference	0.88 (0.73-1.05)	0.16
**Using Data from *Worse Eye***					
**Child Refractive Astigmatism, D[c]**			**Child Corneal Astigmatism, D[d]**		
	OR[a] (95% CI)	*P* Value		OR[a] (95% CI)	*P* Value
Maternal refractive astigmatism, D	0.81 (0.74-0.89)	<0.001	Maternal corneal astigmatism, D	1.58 (1.40-1.79)	<0.001
Paternal refractive astigmatism, D	0.85 (0.78-0.92)	<0.001	Paternal corneal astigmatism, D	1.34 (1.20-1.51)	<0.001
Child's age, years	0.91 (0.83-0.99)	0.03	Child's age, years	1.10 (1.00-1.22)	0.06
Sex, male as reference	0.89 (0.76-1.03)	0.12	Sex, male as reference	0.92 (0.78-1.09)	0.36
Child's spherical equivalent, D	0.83 (0.79-0.88)	<0.001	Child's axial length, mm	0.78 (0.71-0.86)	<0.001
Outdoor time, hours per day	0.92 (0.80-1.07)	0.29	Outdoor time, hours per day	0.91 (0.78-1.06)	0.25
Near work, diopter hours per day	1.00 (0.97-1.03)	0.86	Near work, diopter hours per day	1.03 (1.00-1.07)	0.05

© 2022 Kam KW et al. *JAMA Network Open.*

Family income per month, <HK $20000 as reference	1.05 (0.86-1.28)	0.63	Family income per month, <HK $20000 as reference	1.05 (0.85-1.29)	0.67
Mother education level, without post-secondary as reference	1.04 (0.86-1.25)	0.72	Mother education level, without post-secondary as reference	1.04 (0.86-1.27)	0.69
Father education level, without post-secondary as reference	0.76 (0.63-0.92)	0.004	Father education level, without post-secondary as reference	0.86 (0.71-1.04)	0.13

Abbreviation: D, diopter; OR, odds ratio; CI, confidence interval.

[a] OR was estimated by multiple logistic regression model.

[b] *P*-trend of parental influence was estimated by univariate analysis.

[c] Age, sex, child's spherical equivalent, outdoor time, near work, family income and parental education level were adjusted in refractive astigmatism model.

[d] Age, sex, child's axial length, outdoor time, near work, family income and parental education level were adjusted in corneal astigmatism model.

© 2022 Kam KW et al. *JAMA Network Open.*

eTable 5. Age and Sex Association Analysis on Child Astigmatism in 5708 Trios

**Age (years)**	**Estimated mean RA[a] (D)**	**Estimated mean CA[a] (D)**
6	-0.76 (-0.81 to -0.72)	1.28 (1.24-1.33)
7	-0.71 (-0.75 to -0.67)	1.25 (1.21-1.29)
8	-0.69 (-0.74 to -0.64)	1.22 (1.17-1.27)
P-trend[b]	0.04	0.13
**Sex**		
Male	-0.74 (-0.78 to -0.71)	1.24 (1.21-1.28)
Female	-0.71 (-0.75 to -0.67)	1.27 (1.23-1.31)
P Value[c]	0.18	0.34

[a] Estimated mean values were generated by univariate analyses.

[b] P trend was calculated with the adjustment of parental astigmatism, sex, child's spherical equivalent, outdoor time, near work, family income and parental education level.

[c] P values was calculated with the adjustment of parental astigmatism, age, child's axial length, outdoor time, near work, family income and parental education level.

© 2022 Kam KW et al. *JAMA Network Open.*

eFigure 1. Different Combinations of Parental Astigmatism Severities on Child Astigmatism

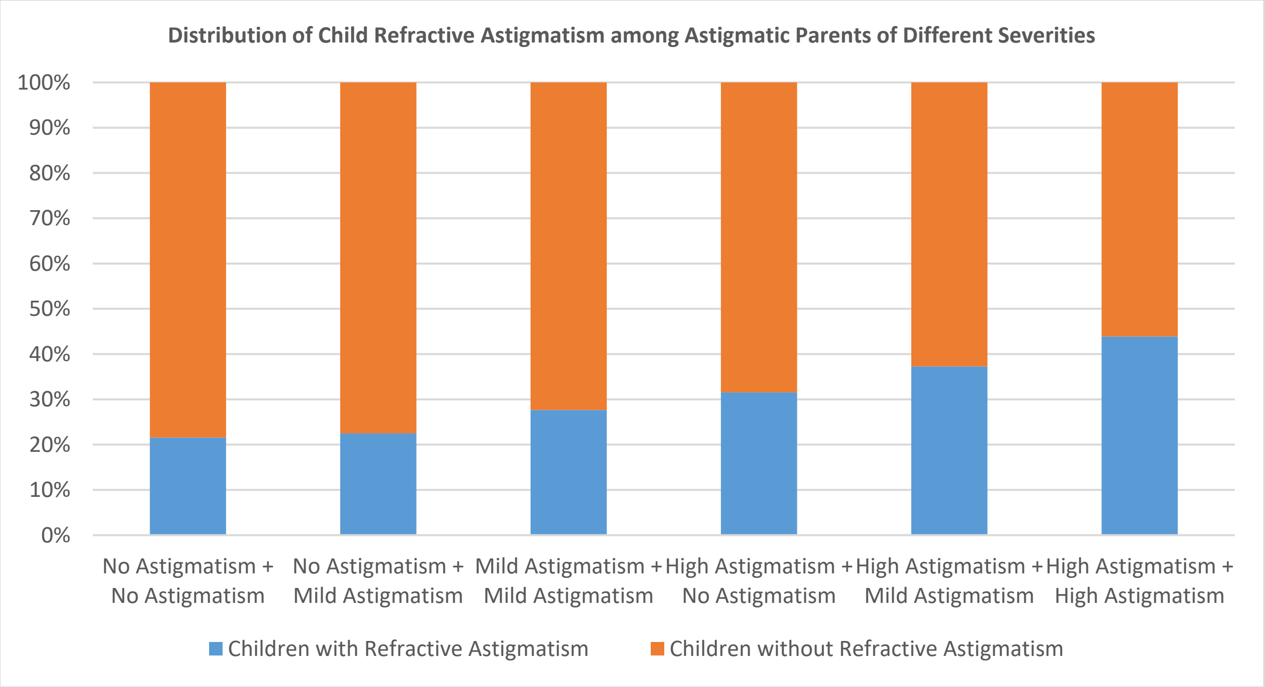

© 2022 Kam KW et al. *JAMA Network Open.*

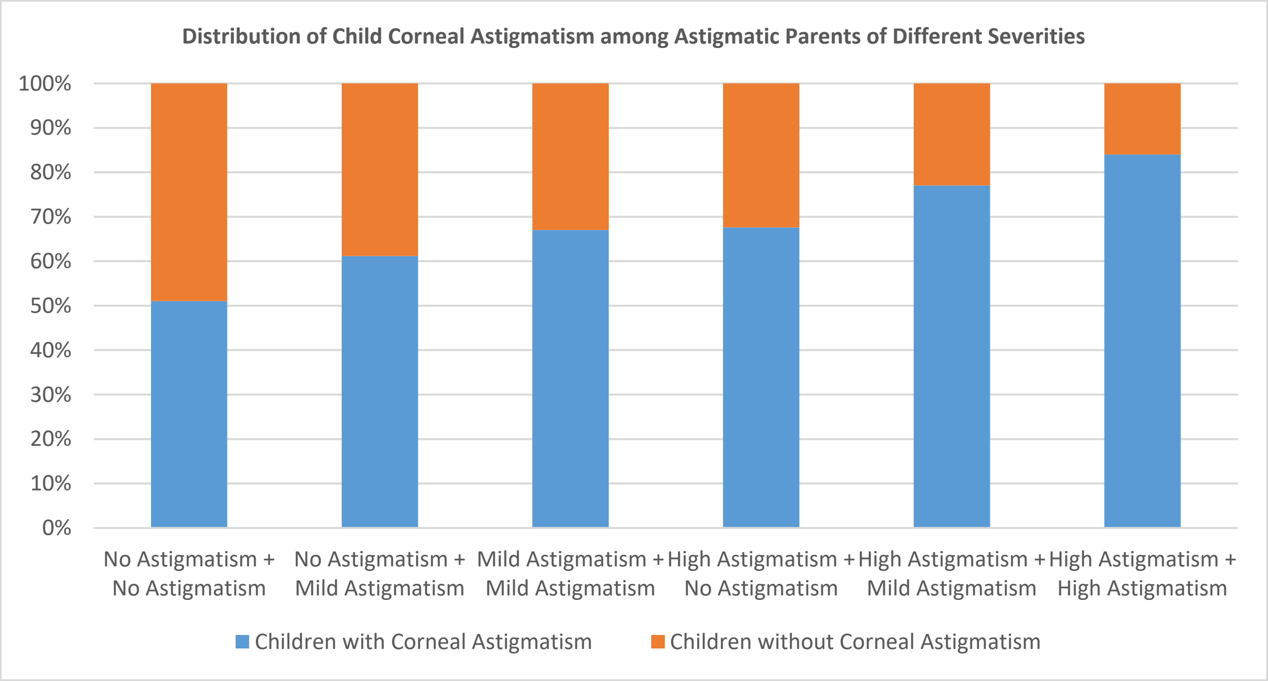

© 2022 Kam KW et al. *JAMA Network Open.*

eAppendix. Hong Kong Children Eye Study Questionnaire

Please tick the average number of ***hours per day*** that your child spends doing the following activities (***e.g. if your child does 15 hours of outdoor leisure activities per week, then the average number of hours per weekday is 3 hours***). If your child spends ***more than 3 hours*** on a particular activity, please specify ***the amount of hours spent***.

	**On a school weekday**				**On a school weekend**				**On a vacation day (e.g. summer and winter break)**			
	Not at all	Less than 1 hour	1-2 hours	3 or more hours	Not at all	Less than 1 hour	1-2 hours	3 or more hours	Not at all	Less than 1 hour	1-2 hours	3 or more hours
a) Out of door activities(in your backyard, walking, riding a bike/scooter)	☐	☐	☐	☐ ___ hours	☐	☐	☐	☐ ___ hours	☐	☐	☐	☐ ___ hours
b) Outdoor leisure activities (*e.g. BBQs, picnic, beach, bushwalk*)	☐	☐	☐	☐ ___ hours	☐	☐	☐	☐ ___ hours	☐	☐	☐	☐ ___ hours
c) Indoor activities (gymnasium, dance studio,)	☐	☐	☐	☐ hours	☐	☐	☐	☐ hours	☐	☐	☐	☐ hours
c) Watching T.V/ videos/ DVDs	☐	☐	☐	☐ ___ hours	☐	☐	☐	☐ ___ hours	☐	☐	☐	☐ ___ hours
d) Playing home video game (e.g. *PlayStation)*	☐	☐	☐	☐ ___ hours	☐	☐	☐	☐ ___ hours	☐	☐	☐	☐ ___ hours
e) Drawing, painting and/or writing	☐	☐	☐	☐ ___ hours	☐	☐	☐	☐ ___ hours	☐	☐	☐	☐ ___ hours
f) Hobbies and crafts	☐	☐	☐	☐ ___ hours	☐	☐	☐	☐ ___ hours	☐	☐	☐	☐ ___ hours
g) Cooking, making or constructing things	☐	☐	☐	☐ ___ hours	☐	☐	☐	☐ ___ hours	☐	☐	☐	☐ ___ hours
h) School homework	☐	☐	☐	☐ ___ hours	☐	☐	☐	☐ ___ hours	☐	☐	☐	☐ ___ hours
i) Reading books for pleasure	☐	☐	☐	☐ ___ hours	☐	☐	☐	☐ ___ hours	☐	☐	☐	☐ ___ hours
j) Playing musical instruments	☐	☐	☐	☐ ___ hours	☐	☐	☐	☐ ___ hours	☐	☐	☐	☐ ___ hours
k) Using a computer	☐	☐	☐	☐ ___ hours	☐	☐	☐	☐ ___ hours	☐	☐	☐	☐ ___ hours
l) Playing ***hand-held*** electronic devices (*e.g.*	☐	☐	☐	☐	☐	☐	☐	☐	☐	☐	☐	☐

© 2022 Kam KW et al. *JAMA Network Open.*

mobile phone, Gameboy, iPad/tablet)				___ hours				___ hours				___ hours
m) Playing with and caring for pets	☐	☐	☐	☐ ___ hours	☐	☐	☐	☐ ___ hours	☐	☐	☐	☐ ___ hours
n) Going shopping	☐	☐	☐	☐ ___ hours	☐	☐	☐	☐ ___ hours	☐	☐	☐	☐ ___ hours
o) Playing chess, cards or board games	☐	☐	☐	☐ ___ hours	☐	☐	☐	☐ ___ hours	☐	☐	☐	☐ ___ hours

© 2022 Kam KW et al. *JAMA Network Open.*

Hong Kong Children Eye Study
Questionnaire

1) What is the highest level of education completed by the ***mother***?

- ☐ Never attended school
- ☐ Primary school
- ☐ High school
- ☐ Associate degree or higher diploma
- ☐ University, CAE or some other tertiary institute degree
- ☐ Higher degree including a Masters or PhD

Total amount of years spent in school:_________________years

2) What is the highest level of education completed by the ***father***?

- ☐ Never attended school
- ☐ Primary school
- ☐ High school
- ☐ Associate degree or higher diploma
- ☐ University, CAE or some other tertiary institute degree
- ☐ Higher degree including a Masters or PhD

Total amount of years spent in school:_________________years

Please tick the appropriate box and answer the following questions in Hong Kong dollars.

3) What's your family income?

- ☐ < $5,000
- ☐ $5,000--10,000
- ☐ $10,000 –11,999
- ☐ $12,000 –14,999
- ☐ $15,000 –19,999
- ☐ $20,000 – 24,999
- ☐ $25,000 –29,999
- ☐ $30,000 – 34,999
- ☐ $35,000 – 39,999
- ☐ $40,000 – 49,999
- ☐ $50,000 – 59,999
- ☐ $60,000 – 79,999
- ☐ $80,000 – 99,999
- ☐ $100,000 –144,999
- ☐ $150,000 –199,999
- ☐ ≥ $200,000

© 2022 Kam KW et al. *JAMA Network Open.*
