# Supplementary material for: Association of Maternal and Paternal Astigmatism With Child Astigmatism in the Hong Kong Children Eye Study
Source: JAMA Netw Open. 2022 Dec 21;5(12):e2247795. doi: 10.1001/jamanetworkopen.2022.47795 (PMC9856878; doi:10.1001/jamanetworkopen.2022.47795)
Supplement: Supplement 2. — Data Sharing Statement [file jamanetwopen-e2247795-s002.pdf]

Data Sharing Statement

Kam. Association of Maternal and Paternal Astigmatism With Child Astigmatism in the Hong Kong Children Eye Study. *JAMA Netw Open*. Published December 21, 2022. doi:10.1001/jamanetworkopen.2022.47795

Data
Data available: No
